# Probing the Effects of Chemical Modifications on Anticoagulant and Antiproliferative Activity of Thrombin Binding Aptamer

**DOI:** 10.3390/ijms26010134

**Published:** 2024-12-27

**Authors:** Antonella Virgilio, Daniela Benigno, Carla Aliberti, Ivana Bello, Elisabetta Panza, Martina Smimmo, Valentina Vellecco, Veronica Esposito, Aldo Galeone

**Affiliations:** Department of Pharmacy, University of Naples Federico II, 80131 Napoli, Italy; antonella.virgilio@unina.it (A.V.); daniela.benigno@unina.it (D.B.); carla.aliberti@unina.it (C.A.); ivana.bello@unina.it (I.B.); e.panza@unina.it (E.P.); martina.smimmo@unina.it (M.S.); vellecco@unina.it (V.V.); galeone@unina.it (A.G.)

**Keywords:** G-quadruplex, thrombin binding aptamer, 8-bromo-2′-deoxyguanosine, locked nucleic acid guanosine, 2′-O-methylguanosine, antiproliferative activity

## Abstract

Thrombin binding aptamer (TBA) is one of the best-known G-quadruplex (G4)-forming aptamers that efficiently binds to thrombin, resulting in anticoagulant effects. TBA also possesses promising antiproliferative properties. As with most therapeutic oligonucleotides, chemical modifications are critical for therapeutic applications, particularly to improve thermodynamic stability, resistance in biological environment, and target affinity. To evaluate the effects of nucleobase and/or sugar moiety chemical modifications, five TBA analogues have been designed and synthesized considering that the chair-like G4 structure is crucial for biological activity. Their structural and biological properties have been investigated by Circular Dichroism (CD), Nuclear Magnetic Resonance (NMR), native polyacrylamide gel electrophoresis (PAGE) techniques, and PT and MTT assays. The analogue TBAB contains 8-bromo-2′-deoxyguanosine (**B**) in G-*syn* glycosidic positions, while TBAL and TBAM contain locked nucleic acid guanosine (**L**) or 2′-O-methylguanosine (**M**) in G-*anti* positions, respectively. Instead, both the two types of modifications have been introduced in TBABL and TBABM with the aim of obtaining synergistic effects. In fact, both derivatives include **B** in *syn* positions, exhibiting in turn **L** and **M** in the *anti* ones. The most appealing results have been obtained for TBABM, which revealed an interesting cytotoxic activity against breast and prostate cancer cell lines, while in the case of TBAB, extraordinary thermal stability (T_m_ approximately 30 °C higher than that of TBA) and an anticoagulant activity higher than original aptamer were observed, as expected. These data indicate TBAB as the best TBA anticoagulant analogue here investigated and TBABM as a promising antiproliferative derivative.

## 1. Introduction

During the last 25 years, a great plethora of nucleotide derivatives have been developed and the effects of their introduction in DNA and RNA sequences as potential therapeutic oligonucleotides have been evaluated in vitro and often in vivo [1,2,3,4,5,6,7]. Since the first investigations in this frame, it has been evident that the chemical modification of oligonucleotides (ODNs) is a critical feature to the accomplishment of their therapeutic application, often resulting in significant improvements in many properties. Indeed, the way to successful oligonucleotide drugs needs to overcome many hurdles, such as their poor stability both from a thermodynamic point of view and in biological environments, in which degradation is favored by exo- and endonucleases [8].

Furthermore, chemical modifications aimed at improving target site accessibility and affinity, delivery, and biodistribution, i.e., binding to proteins and receptors, as well as toxicity and complement activation, are also of particular concern [9,10,11,12]. Because of their outstanding affinity and specificity toward a given target, and high potential in diagnostic and therapeutic applications [13,14], over and above in bioengineered constructs [15,16,17], aptamers are one of the most studied categories of protein ligands.

A fair number of this class of therapeutic and diagnostic oligonucleotides folds in G-quadruplexes (G4-aptamers), since these peculiar DNA/RNA conformations are frequently characterized by extraordinary thermal stabilities and the secondary structure of aptamers is essential for their functions [3,18]. G-rich oligonucleotides (GROs) can adopt G-quadruplex structures (G4s) under physiological conditions through Hoogsteen hydrogen bonds [19,20], and G4s are very rife in the human genome, remarkably in promoter regions and telomere ends [21,22,23,24,25].

Quite recently, through cell-based assays, it was demonstrated that some GROs can selectively behave as antiproliferative agents [26,27,28,29]. An interesting example of this class of oligonucleotides is the 26-mer AS1411 (a guanine-rich G4-forming DNA aptamer) which exhibits growth-inhibitory properties against a broad range of cancer cell lines in vitro [30,31] and can act as a new nanodrug delivery system for targeted delivery to improve the cancer therapy efficacy [32,33]. The AS1411 therapeutic potential is mostly associated with its capability to be taken up by the target cells due to its interaction with cell surface nucleolin [34], a protein over-expressed in cancer cells. Unfortunately, the 26-mer AS1411, as with most of the anticancer GROs, is structurally polymorphic [35,36,37], adopting in solution many G4 conformations characterized by different molecularity and/or loop arrangement [38]. Nevertheless, to have an adequate understanding of the structure–activity relationships of potential therapeutic ODNs, the availability of biologically active sequences forming single and well-defined G4s is crucial.

**Figure 1:**
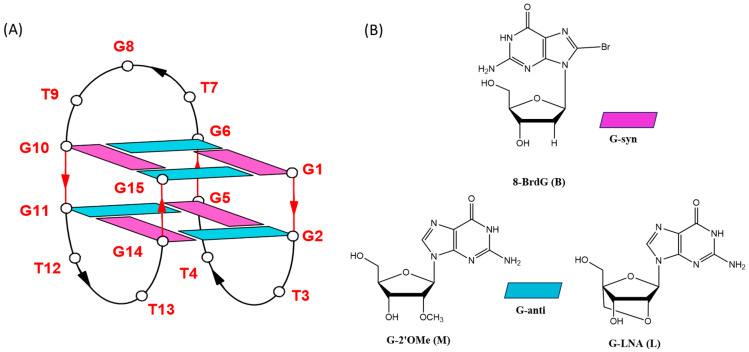
Schematic representation of the TBA G-quadruplex. Deoxyguanosines in *syn* and *anti* glycosidic conformations are in purple and light blue, respectively (**A**); chemical structures of 8-bromo-2′-deoxyguanosine (**B**) (inserted into the G-*syn* positions of the original sequence), 2′-O-methylguanosine (**M**), and locked nucleic acid guanosine (**L**) (inserted into the G-*anti* positions of the original sequence) (**B**).

Among the cell-based screened GROs, a 15-mer ODN of sequence G_2_T_2_G_2_TGTG_2_T_2_G_2_, known as TBA (thrombin binding aptamer), turned out to possess an interesting antiproliferative activity, in addition to remarkable anticoagulant properties [26,39,40,41]. TBA exhibits some non-negligible advantageous features, such as, principally, its reasonably small size and its ability to fold in a well-defined G-quadruplex structure. The G4 conformation assumed by TBA in K^+^ buffer resembles a chair by adopting an antiparallel monomolecular strand orientation with two stacked G-tetrads connected through one large TGT and two small TT loops (Figure 1) [39,40,41]. After its discovery, TBA has undergone several chemical modifications planned to increase thermal stability and nuclease resistance in biological environments, as well as to improve anticoagulant activity [42,43,44].

Synthetic nucleotide analogues can be properly used to replace canonical residues involved in the arrangement of guanine tetrads, loops, and sugar and phosphodiester linkages [43,45,46], considering that the stacked G-quartets, forming the G4 aptamer core, are mainly responsible for thermal stability, while the loop residues usually perform a key function in the target protein interaction and sugar-phosphate backbone variations can influence the biological resistance. Both modifications involving the sugar-phosphate backbone and the substitution of single bases in the loops have given valuable information about the role of each residue in the structural stability and/or anticoagulant properties [47,48,49,50,51]. Latterly, on the contrary, Svetlova et al. examined the effects of modification in the G4 core of TBA on its ability to interact with thrombin by using alpha-2′-deoxyguanosine and 8-bromo-2′-deoxyguanosine to alter *syn*–*anti* conformations of particular dG-residues [52]. Their data suggest that core guanines not only support the stability and the adoption of a peculiar G4 topology [53], but also set structural parameters for functional recognition of the protein surface, as also confirmed by Yan Xu et al. and some of us in recent studies [54,55].

Indeed, in our paper, we demonstrated that two TBA analogues, which contain one and two extra G-tetrads (namely TBAG3 and TBAG4) and adopt a parallel G4-structure and an “expanded” antiparallel “chair-like” G4 similar to TBA, respectively, do not show anticoagulant activity, probably since the not canonical topology, in the first case, and the larger size of G-core, in the second one, prevent an appropriate interaction with thrombin [54]. However, in view of the potential development of TBA derivatives as antiproliferative agents, the absence of anticoagulant properties should not be thought as a drawback.

In this frame, in one of our latest papers, we have also studied three TBA variants with modified G-tetrads to assess the impacts of nucleobase and sugar moiety chemical modifications on nuclease resistance and anticoagulant properties, yet conserving the canonical chair-like G4 conformation of parent TBA with two G-tetrads [56]. Particularly, three TBA analogues have been investigated, in which 8-bromo-2′-deoxyguanosine (**B**) and locked nucleic acid guanosine (**L**), 2′-O-methylguanosine (**M**) or 2′-F-riboguanosine (**F**) (the last three modification being usually used in therapeutic aptamers to improve nuclease resistance) have been appropriately introduced in *syn* and *anti* positions of the TBA G-quadruplex central core, taking into account that **B** promotes the *syn*-glycosidic conformation while **L**, **M** and **F** the *anti* one. The whole of the data has revealed TBABF as the analogue endowed with the highest anticoagulant activity. Interestingly, although all modified TBAs have shown increased thermal and nuclease stability, the anticoagulant properties of TBABL and TBABM have turned out insignificant or lower than those of the parent counterpart, thus being totally powerless to inhibit thrombin [56], as previously observed for other TBA analogues [57]. Considering that the simultaneous anticoagulant activity of TBA corresponds to a disadvantage in developing its derivatives as anticancer agents [58,59], we decided to test the antiproliferative potential of TBABL and TBABM as examples of the synergic effect of different chemical modifications, and TBAB, TBAL, and TBAM as new TBA derivatives characterized by a single type of substitution at time. In particular, although the effect of **B** introduction in the TBA sequence has already been tested on the anticoagulant activity of this aptamer [60], to the best of our knowledge, the combined effect of the simultaneous substitution of four dG canonical residues with **B** in G-tetrad *syn* positions, as in TBAB, on thermal stability and anticoagulant activity has never been evaluated.

Therefore, in an effort to further continue our previous research, by testing the effects of some combinations of three different chemical modifications on both the anticoagulant and antiproliferative activity of TBA, as well, these new TBA analogues containing B in all *syn* G-tetrad positions (TBAB), or **L** or **M** in all *anti* G-tetrad ones (TBAL and TBAM, respectively) (Figure 1, Table 1) have been investigated by CD, NMR, and non-denaturing PAGE techniques, and their biological properties have been evaluated and compared with those of their natural counterpart, and derivatives TBABL and TBABM [56].

## 2. Results

### 2.1. Structural Insights of Investigated TBA Derivatives

To verify the ability of these modified sequences to adopt G-quadruplex (G4) structures and to have evidence concerning their folding topologies, the TBA analogues in Table 1 were examined by CD, 1H NMR, and non-denaturing PAGE techniques.

#### 2.1.1. Analysis of CD Spectra

**Figure 2:**
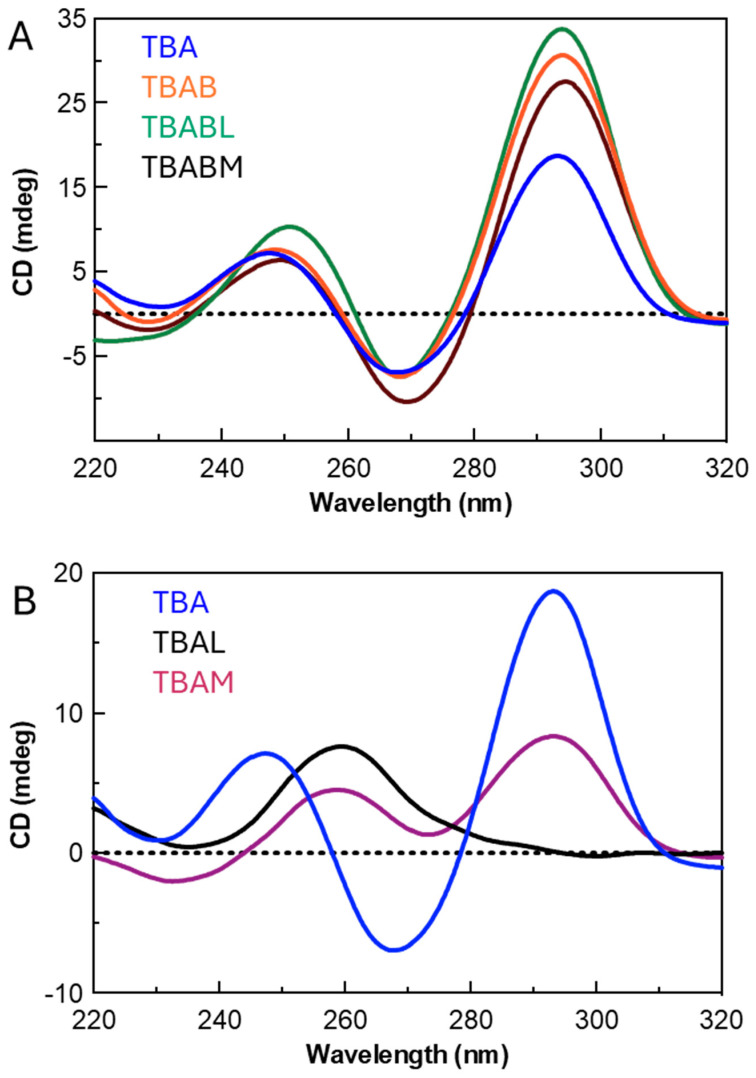
CD spectra at 37 °C of TBA and its studied analogues. Panel (**A**): TBA, TBAB, TBABL, TBABM; Panel (**B**): TBA, TBAL, TBAM.

To confirm the folding topology of native TBA and its analogues at 37 °C in a potassium buffer, their CD spectra were recorded (Figure 2) [61]. As expected, the natural counterpart exhibited the characteristic CD profile of antiparallel G4s with two positive bands around 245 and 295 nm, and a negative one around 265 nm [61,62,63]. In the same experimental conditions, TBAB, TBABL, and TBABM showed CD spectra almost identical to the unmodified TBA (Figure 2A), suggesting they adopt the same G4 folding topology. Instead, TBAL showed a CD profile with a maximum at 264 nm and a minimum at 240 nm, being typical of parallel-stranded G4s with all the guanosines of the tetrads in *anti*-glycosidic conformation (Figure 2B). Differently to the previous cases, TBAM displayed a mixed CD profile with two positive bands around 295 nm and 260 nm, indicative of the presence in solution of different structured species, supposedly including an antiparallel G4 with alternating G-*syn* and G-*anti* and a parallel G4 with all the Gs in *anti* (Figure 2B). Furthermore, the intensity of the CD peaks of TBAL and TBAM was lower than that of the natural counterpart, probably due to their partial unfolding at 37 °C in the previously specified buffer, thus suggesting the inability of these TBA analogues to form very stable G4s.

#### 2.1.2. Thermal Stability Analysis

To evaluate the thermal stability of modified ODNs under the same experimental conditions previously described, the CD melting curves were recorded for all derivatives and melting temperatures (T_m_) were consequently determined (Figure S1). The T_m_ values are reported in Table 1. The elevated T_m_ value of TBA derivatives containing **B** in *syn* position of the original sequence, namely TBAB, TBABL, and TBABM, revealed that this chemical modification contributes significantly to the formation of stable G-quadruplexes [64], since TBAB, which contains only this type of modification, showed a T_m_ about 30 °C higher than its natural counterpart. Instead, by confirming the CD spectra data, TBAM and TBAL form less stable G-quadruplexes compared to TBA, thus accounting for their partial unfolding at 37 °C. Data obtained from heating/cooling CD profiles indicated that, from the combination of chemical modifications increasing or decreasing thermal stability, in the cases TBABL and TBABM, the stabilizing effect of **B** predominates over the destabilizing effect of **M** and **L**, thus guaranteeing the formation of structures with T_m_ approximately 20 °C higher than that of the natural sequence. Furthermore, similarly to the unmodified aptamer, derivatives containing **B** were characterized by a reversible transition in denaturation/renaturation processes, as indicated by the almost superimposable CD melting and annealing curves [65]. The absence of hysteresis between the heating and cooling profiles suggested the existence of single well-defined monomolecular G-quadruplex structures with relatively fast folding/unfolding kinetics. On the contrary, TBAL and TBAM showed an evident hysteresis between melting and annealing curves, thus indicating that their folding and unfolding processes are not at thermodynamic equilibrium. These data are compatible with the presence in solution of a G4 differing from that formed by the natural sequence, in the case of TBAL, or more than one G-quadruplex complexes, in the case of TBAM, as already suggested by CD spectra (Figure S1) [65,66,67].

#### 2.1.3. Native Polyacrylamide Gel Electrophoresis (PAGE)

**Figure 3:**
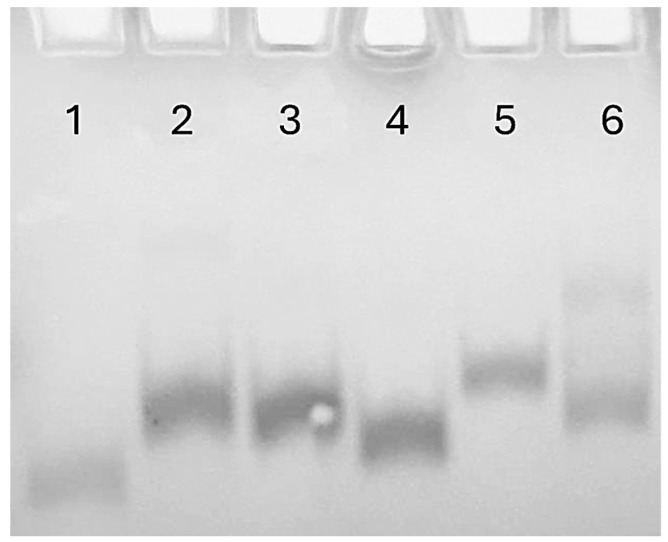
PAGE analysis of TBA and its investigated analogues. Lane 1: TBA; lane 2: TBABM; lane 3: TBABL; lane 4: TBAB; lane 5: TBAL; lane 6: TBAM. See Section 4 for experimental details.

Native polyacrylamide gel electrophoresis (PAGE) was performed to confirm the ability of the studied derivatives to fold or not into a single G-quadruplex structure strictly similar to the parent one, since the G4 electrophoretic mobility is influenced both by folding topology and molecularity [68] (Figure 3). Despite slightly slower migration rates attributable to their higher masses than TBA (used as reference), TBAB, TBABL, and TBABM showed electrophoretic profiles that confirmed their ability to fold in a monomolecular compact structure very similar to that of the original sequence. TBAL also exhibited a single well-defined bandwidth electrophoretic motility, thus indicating that it folds in a single G4 structure. However, this structure has a slower mobility than the derivatives with higher molecular weight (namely, TBABM and TBABL) thus indicating the involvement of other factors able to decrease motility. In agreement with what was suggested by the CD profile, this datum is consistent with the presence of a less compact parallel G-quadruplex structure characterized by three propeller loops. By contrast, the TBAM lane is characterized by two bands with different electrophoresis motility, in particular, a migrating band with mobility comparable to TBA and **B** containing analogues supposedly ascribable to an antiparallel TBA-like G4, and a lower migrating band compatible with a parallel G4 with three propeller loops. These data confirmed the presence in solution of two different G4-complexes, as suggested by the TBAM CD profile.

#### 2.1.4. Nuclear Magnetic Resonance (1H-NMR)

**Figure 4:**
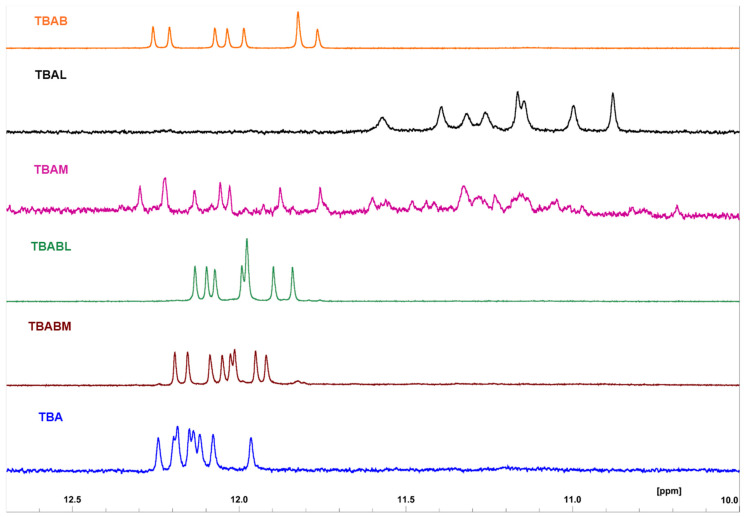
Imino proton regions of 1H-NMR spectra (700 MHz) of TBA and its investigated analogues.

To reinforce structural insight about the investigated TBA derivatives, 1H NMR experiments were also assessed. 1H NMR spectra (Figure 4) revealed that most derivatives form a single well-defined G-quadruplex, as indicated by the presence of eight signals in the region between 12.5–10.5 ppm attributable to imino protons involved in the formation of two G-tetrads [69,70,71]. Particularly, the imino proton regions of TBA analogues containing **B** are closely similar to each other and to their natural counterpart, besides slight differences in chemical shifts mainly ascribable to the presence of the bromine atom. Data suggested that these derivatives, despite the chemical modifications, preserve the topology of the original structure. In the case of TBAL, the 1H NMR spectrum in the imino proton region, considered diagnostic of the presence of G4, showed the same number of signals as TBA, although with completely different chemical shift values. These data confirmed the aptitude of TBAL to adopt a G4 conformation different from the natural reference sequence. Finally, the one-dimensional proton spectrum of TBAM revealed the presence of two species in solution, one of which is probably very similar to the natural counterpart, as clearly indicated by the presence of eight well-resolved signals in the downfield imino region, similar to those observed in the TBA 1H NMR spectrum. These data are in good agreement with the structural features outlined by the CD and PAGE experiments.

### 2.2. Antiproliferative Activity

**Figure 5:**
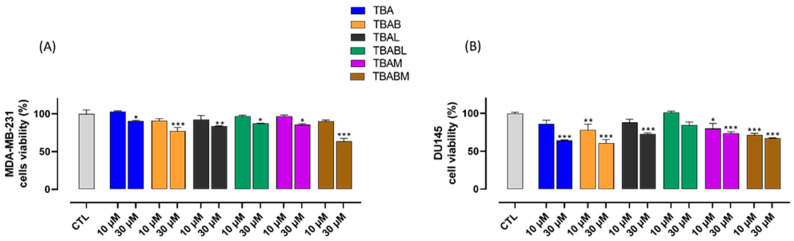
Effect of TBA and its analogues on MDA-MB-231 (Panel **A**) and DU145 (Panel **B**) cell proliferation. Cell proliferation was measured using an MTT assay and evaluated at 48 h. Each experiment (*n* = 3) was run in quadruplicate. * *p* < 0.05; ** *p* < 0.01; *** *p* < 0.001 vs. CTL.

The potential anticancer activity of TBA and its analogues was examined regarding the proliferative capacity of MDA-MB-231 and DU145 cells, two validated in vitro models of human breast and prostate cancer, using MTT assay. Cells were exposed to TBA and its five distinct derivatives. Each compound was tested at 10 and 30 µM for 24 and 48 h. Cells not exposed to the investigated derivatives were used as control. Our results demonstrated that both TBA and its analogues, at a concentration of 30 µM after 48 h, exhibited antiproliferative effects on MDA-MB-231 and DU145 cells. However, only TBAB, TBAM, and TBABM at 10 µM significantly inhibited the proliferation of DU145 cells (Figure 5A,B). Similar results were also obtained at 24 h (Figure S2).

### 2.3. Anticoagulant Activity

**Figure 6:**
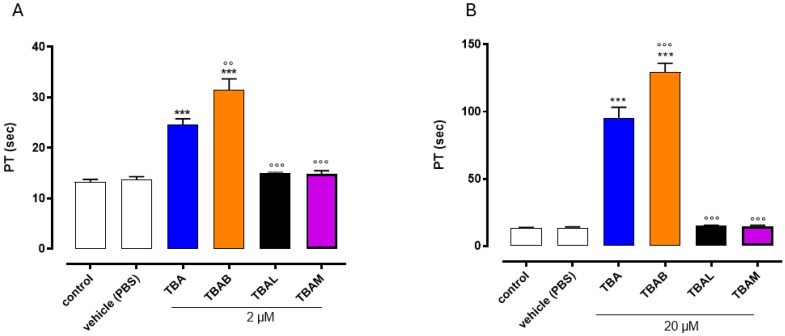
Effect of TBA and its analogues on PT assay. PT values of modified TBAs and natural counterpart were evaluated at 2 µM (Panel **A**) and 20 µM (Panel **B**). *** *p* < 0.001 vs. vehicle, ◦◦ *p* < 0.01, ◦◦◦ *p* < 0.001 vs. TBA.

To assess the anticoagulant property of modified TBA analogues, ODNs were tested using a PT assay, and their activity was compared to the original TBA. The results revealed that the TBAM and TBAL exhibited no anticoagulant activity at both the concentrations tested (2–20µM) (Figure 6A,B). Conversely, TBAB displayed a significantly increased PT value at both concentrations used, compared to its natural counterpart TBA, thus indicating a noteworthy anticoagulant property (Figure 6A,B). Specifically, at the highest concentration tested (20 µM), the TBAB-induced PT time was about 30% longer than the unmodified TBA (129.5 ± 6.5 s vs. 95.0 ± 8.2 s for TBAB and TBA, respectively). Similar results were also obtained at 2µM (31.5 ± 2.1 s vs. 24.6 ± 1.2 s for TBAB and TBA, respectively).

### 2.4. Nuclease Stability Assay

The stability of nucleic acid aptamers is a hot point for their potential use in therapeutic applications. Therefore, to evaluate the resistance in biological environments, all investigated ODNs were tested in a degradation assay in fetal bovine serum (FBS) and analyzed at different times by CD (Figure S3) [72]. To check the persistence over time of the CD signal of undegraded G-quadruplex structures in solutions, CD spectra of all ODNs were registered in the range 0–72 h at 37 °C in 10% FBS. Most of the analyzed aptamers showed CD signals decreasing in a time-dependent manner. CD spectra of TBAL and TBAM were acquired at different time points than the **B** containing analogues, already tested in 50% FBS in the range 0–24 h, revealing an interesting nuclease stability until 4 h (50% undegraded species in solution). TBAL and TBAM, in experimental conditions used here, resulted almost completely degraded already at 1 h (Figure S3), differently from TBABL and TBABM that exhibited similar partial resistance to nucleases, since 30–40% of G-quadruplex species were still present at 24 h, in both cases. The most interesting results were obtained for TBAB, which revealed a noteworthy improvement in nuclease resistance in comparison to the other analogues, since its CD band intensity at 295 nm decreases by about 50% at 24 h, 60% at 48 h and 85% at 72 h.

## 3. Discussion

The development of a mere nucleic acid sequence into a therapeutic oligonucleotide often entails steps of trial-and-error chemical modifications aimed at improving the general properties of the unmodified oligonucleotide. This is especially true in the case of aptamers whose structural characteristics are often critical for the appropriate interaction with the target and a relevant biological activity. Among GRO endowed with antiproliferative properties, often characterized by a misleading polymorphism, TBA represents a special case since it adopts a well-defined G4 chair-like conformation that simplifies the analysis of the effect of chemical modifications on the structural features. In this paper, we have investigated the properties of TBA derivatives containing 8-bromo-2′-deoxyguanosine (**B**), locked nucleic acid guanosine (**L**), or 2′-O-methylguanosine (**M**) (Table 1), with the last two nucleoside analogues often inserted in the sequence of therapeutic oligonucleotides.

CD, PAGE, and NMR data clearly indicate that the presence of **B** in *syn* positions is a critical feature in preserving the original TBA conformation since only TBAB, TBABL, and TBABM fold in a unique G4 chair-like structure. This feature is also important in increasing thermal stability, as suggested by CD melting data showing definitively higher T_m_ for TBA analogues containing **B**, as expected based on data already reported in the literature. It is notable that such straightforward chemical modification in TBAB causes a remarkable increase of thermal stability (Tm approximately 30 °C higher than that of TBA), compared to the unmodified aptamer. In fact, previous studies reported in the literature [60,64] have explored the effects of simultaneously introducing multiple **B** residues into the TBA sequence. However, to the best of our knowledge, the specific substitution of all dG *syn* canonical residues within the G-tetrad core with 8-bromo-2′-deoxyguanosine, as in TBAB, has never been evaluated. Moreover, no case has demonstrated a melting point increase comparable to that observed for TBAB. Thus, this notable surplus in terms of thermodynamic stability is probably attributable to the additional stacking effect that the bromine substituent may induce in modified G4s.

In addition to the interesting new results concerning the thermal stability of TBAB, it is noteworthy that the aim of the present paper is also to identify new antiproliferative aptamers and to test for the first time the synergistic effects of two chemical modifications at G-core level on the antiproliferative activity of TBA, to improve the applicative potential of TBA in this therapeutic field. Therefore, the antiproliferative properties of all TBA analogues here investigated were tested on two cell lines, namely MDA-MB-231 and DU145 (human breast and prostate cancer, respectively) with different aptamer concentrations and exposition times. The decreasing order of activity in MDA-MB-231 cell line for the 48 h treatment is approximately TBABM ≥ TBAB ≥ TBAL > TBAM ≥ TBABL > TBA for both the tested concentrations, while the same decreasing order appears slightly different in DU145 cell line depending on aptamer concentration, being TBABM > TBAB > TBAM > TBA > TBAL > TBABL for 10 µM concentration and TBAB > TBA > TBABM > TBAM ≈ TBAL > TBAL for 30 µM concentration. Results indicate that TBABM and TBAB are the most active TBA analogues in both cancer cell lines. These data also suggest that the presence of a highly stable G4 chair-like conformation (as for TBABM and TBAB) would seem very important for the antiproliferative activity. However, this structural characteristic cannot be considered the only one required to have an antiproliferative activity since TBABL, which also folds in a very stable G4 chair-like structure, shows only a negligible antiproliferative activity, thus highlighting the importance of the local conformation of the aptamer beside the general one for the antiproliferative activity.

Furthermore, in the present study, the anticoagulant properties of three TBA derivatives (namely, TBAB, TBAL, and TBAM) have been evaluated, while for TBABM and TBABL the same biological activity was reported in a previous paper [56]. PT measurements indicate that TBAL and TBAM show no anticoagulant activity, while TBAB is endowed with an anticoagulant activity far higher than that of the native aptamer TBA. Unfavorable data concerning TBAL and TBAM were expected to a certain extent, considering that they are unable to fold in a dominant G4 chair-like conformation which, in several papers, has proven to be fundamental for interacting with thrombin and inducing an anticoagulant activity [41,52]. On the other hand, TBABM and TBABL only showed a reduced anticoagulant activity by far lower than that of the original aptamer TBA [56].

In summary, the main results of our investigation are: (1) the improvement of the anticoagulant activity of the original aptamer by introducing only **B** residues (namely TBAB) and (2) the development of a promising antiproliferative TBA analogue (namely TBABM, containing both **B** and **M** residues) with a negligeable anticoagulant activity.

## 4. Materials and Methods

### 4.1. Oligonucleotide Synthesis and Purification

The ODNs listed in Table 1 were synthesized by an ABI 394 DNA synthesizer using solid-phase β-cyanoethyl phosphoramidite chemistry at 10 µmol scale. The synthesis was carried out using normal 3′-phosphoramidites (Link Technologies, Glasgow, UK). The modified monomers were introduced in the sequences using commercially available 5′-dimethoxytrityl-N2-dimethylaminomethylidene-8-bromo-2′-deoxyGuanosine, 3′-[(2-cyanoethyl)-(N,N-diisopropyl)]-phosphoramidite; 5′-dimethoxytrityl-N-dimethylformamidine-(2′-O, 4′-C methylene)Guanosine, 3′-[(2-cyanoethyl)-(N,N-diisopropyl)]-phosphoramidite; and 5′-dimethoxytrityl-N-isobutyryl-Guanosine, 2′-O-methyl, 3′-[(2-cyanoethyl)-(N,N-diisopropyl)]-phosphoramidite (Glen Research, Sterling, VA, USA). For all ODNs, a universal support was used. The oligomers were detached from the support and deprotected by treatment with concentrated aqueous ammonia at room temperature for 24 h. The combined filtrates and washings were concentrated under reduced pressure, redissolved in H_2_O, analyzed, and purified by high-performance liquid chromatography on a Nucleogel SAX column (Macherey-Nagel, Duren, Germany, 1000-8/46) using buffer A 20 mM NaH_2_PO_4_/Na_2_HPO_4_ aqueous solution (pH 7.0) containing 20% (*v*/*v*) CH_3_CN) and buffer B (1 M NaCl and 20 mM NaH_2_PO_4_/Na_2_HPO_4_ aqueous solution (pH 7.0) containing 20% (*v*/*v*) CH_3_CN). A linear gradient from 0% to 100% B for 45 min and a flow rate of 1 mL/min were used. The fractions of the oligomers were collected and successively desalted using Sep-Pak cartridges (C-18). The isolated oligomers proved to be >98% pure by HPLC (Macherey–Nagel, 1000-8/46, buffer A: 20 mM NaH_2_PO_4_/Na_2_HPO_4_ aqueous solution (pH 7.0) containing 20% (*v*/*v*) CH_3_CN; buffer B: 1 M NaCl, 20 mM NaH_2_PO_4_/Na_2_HPO_4_ aqueous solution (pH 7.0) containing 20% (*v*/*v*) CH_3_CN; linear gradient from 0 to 100% B for 45 min and flow rate 1 mL/min) (Figure S4), and by NMR (Figure S5).

### 4.2. CD Spectroscopy

CD samples of the oligonucleotides reported in Table 1 were prepared at an ODN concentration of 50 µM using a potassium phosphate buffer (10 mM KH_2_PO_4_/K_2_HPO_4_ and 100 mM KCl, pH 7.0) and submitted to the annealing procedure (heating at 90 °C and slowly cooling at room temperature). The CD spectra of all quadruplexes and CD melting/annealing curves were registered on a Jasco 715 CD spectrophotometer (Jasco, Tokyo, Japan). For the CD spectra, the wavelength was varied from 220 to 320 nm at a 100 nm min^−1^ scan rate, the spectra recorded with a response of 4 s, at 1.0 nm bandwidth and normalized by subtraction of the background scan with buffer. The temperature was kept constant at 37 °C with a thermoelectrically controlled cell holder (Jasco PTC-348). CD melting and annealing curves were registered as a function of temperature (range: 20–95 °C) for all G-quadruplexes, annealed as previously reported, at their maximum Cotton effect wavelengths. The CD data were recorded in a 0.1 cm pathlength cuvette with a scan rate of 30 °C/h.

### 4.3. Gel Electrophoresis

All oligonucleotides were analyzed by native PAGE. All oligonucleotide samples were prepared at an ODN concentration of 50 µM by using a potassium phosphate buffer (10 mM KH_2_PO_4_/K_2_HPO_4_, 100 mM KCl, pH 7.0) and submitted to the annealing procedure (heating at 80 °C and slowly cooling at room temperature). Each oligonucleotide was loaded onto a 20% polyacrylamide gel containing Tris–Borate-EDTA (TBE) 2.5× and 20 mM KCl. The run buffer was TBE 1× containing 50 mM KCl. For all samples, a solution of glycerol/TBE 10× was added just before loading. Electrophoresis was performed at 8 V/cm at a temperature close to 10 °C. Bands were visualized by UV shadowing.

### 4.4. NMR Spectroscopy

NMR samples were prepared at a concentration of approximately 1 mM in 0.6 mL (H_2_O/D_2_O 9:1 *v*/*v*) of buffer solution with 10 mM KH_2_PO_4_/K_2_HPO_4_, 100 mM KCl, and 0.2 mM EDTA (pH 7.0). All samples were heated for 5–10 min at 90 °C and slowly cooled (10–12 h) to room temperature. The solutions were equilibrated for several hours at 4 °C. The annealing process was assumed to be complete when the 1H NMR spectra were superimposable on changing time. NMR spectra were recorded at 37 °C by employing a 700 MHz Bruker spectrometer (Bruker-Biospin, Billerica, MA, USA). Proton chemical shifts were referenced to the residual water signal, resonating at 4.63 ppm (37 °C, pH 7.0). Water suppression was achieved using the excitation sculpting with the gradient routine included in the “zgesgp” pulse sequence [73]. NMR data processing was performed by using the vendor software TOPSPIN 4.1.4 (Bruker Biospin Gmbh, Rheinstetten, Germany).

### 4.5. MTT Assay

Human breast cancer cell line MDA-MB-231 (cat. no. HTB-26) and human prostate cancer cell line DU145 (cat. no. HTB-81) were acquired from the American Type Culture Collection (ATCC, Manassas, VA, USA). Cells were cultured in DMEM (Sigma-Aldrich, Milan, Italy; cat. no. D6546) supplemented with 10% fetal bovine serum (FBS) (Gibco, Milan, Italy; cat. no. A4736301), penicillin (100 U/mL), and streptomycin (100 μg/mL) (cat. no. 30-002-CI), 2 mmol/L L-glutamine (cat. no. 25-005-CI), and 0.01 M HEPES buffer (cat. no. 25-060-CI) (all from Corning, Manassas, VA, USA), and placed at 37 °C in a humidified incubator containing 5% CO_2_. Cells were seeded into 96-well plates (3 × 10^3^ cells/well) and adhered overnight. Cells were treated with TBA, TBAB, TBAL, TBABL, TBAM, and TBABM (10 and 30 µM) for 24 and 48 h. A volume of 100 μL/well of MTT (3-(4,5-dimethylthiazol-2-yl)-2,5-diphenyltetrazolium bromide, cat. M5655, Merk, Italy) (final concentration 0.25 mg/mL in DMEM) was added and incubated for 3 h at 37 °C. After this time, MTT was removed, and the formed purple formazan crystals were dissolved in 100 μL/well DMSO. The absorbance was measured at 540 nm by a microplate spectrophotometer reader (Thermo Scientific Multiskan GO, Thermo Fisher Scientific, Waltham, MA, USA).

### 4.6. Prothrombin Time (PT)

PT assay was conducted on human plasma using the Start Max analyzer (Stago) and a specific kit called neoplastine Cl plus (Stago, Asnieres sur Seine, France). Two levels of human control (STA Coag Control N and P) were used for daily quality control assessments and analytical performance evaluations. Reagents and controls were reconstituted according to the manufacturer’s instructions. This method relies on the high sensitivity of thromboplastin reagent based on recombinant human tissue factors. In the presence of calcium ions, the addition of neoplastine to the plasma initiates the activation of the extrinsic pathway that leads to the conversion of fibrinogen into fibrin and the formation of a solid gel. In our experimental conditions, each ODN or vehicle was incubated with 50 μL of plasma at 37 °C for 15 min. Then, 100 μL of the kit solution containing neoplastine was added, resulting in the activation of the extrinsic pathway. Specifically, the evaluation of PT at the concentration of 20 μM was performed by adding 1 μL of the ODN solution (1 mM) or vehicle (phosphate buffer saline, PBS) to the microtube. To evaluate PT at 2 μM, 1 μL of a diluted solution (0.1 mM ODN solution in PBS buffer) was added to the microtube. The PT measurement was almost produced in triplicate and the average and the standard error values were calculated and expressed in seconds. The basal clotting time was evaluated by measuring the clotting time in the presence of the vehicle.

### 4.7. Nuclease Stability Assay

Nuclease stability assay of all ODNs was conducted in 10% fetal bovine serum (FBS) diluted with Dulbecco’s Modified Eagle’s Medium (DMEM) at 37 °C and studied by CD analysis. Approximately 14 nmol of stock solution of each ODN (~2 O.D.U.) was evaporated to dryness under reduced pressure, and then incubated with 500 μL 10% FBS at 37 °C. The degradation patterns were analyzed by monitoring the CD signal decrease in each sample at 37 °C, as a function of time. CD spectra at different times for each sample were recorded at 37 °C using a Jasco 715 spectrophotometer equipped with a Peltier temperature control system (Jasco, Tokyo, Japan). Data were collected from 240 to 320 nm with a 1 s response time and a 1 nm bandwidth using a 0.1 cm quartz cuvette. Each spectrum shown is corrected for the spectrum of the reaction medium (10% FBS in DMEM).

## 5. Conclusions

We focused on the effects of the introduction of nucleoside analogues on thrombin binding aptamer biological activity, considering that structural characteristics are generally crucial for it. To estimate the impact of nucleobase and/or sugar moiety chemical modifications, five TBA derivatives exhibiting 8-bromo-2′-deoxyguanosine (**B**) in G-*syn* glycosidic positions (TBAB), locked nucleic acid guanosine (**L**), or 2′-O-methylguanosine (**M**) in G-*anti* ones (TBAL and TBAM, respectively), or both types of modifications (TBABL and TBABM) were designed and synthesized. The structural data obtained by CD, 1H-NMR, and native PAGE techniques revealed that just TBAB, TBABL, and TBABM can adopt a unique G4 chair-like structure, showing that the occurrence of **B** in syn positions is pivotal for the original TBA conformation preservation, also contributing favorably to the thermal stability of these G-quadruplexes. Their antiproliferative activities against MDAMB 231 and DU 145 cancer cell lines and anticoagulant properties were analyzed in comparison with TBA. The most interesting results were obtained for TBAB and TBABM, both exhibiting remarkable thermal stability and a chair-like G4 structure. Thus, as far as biological properties, TBAB showed an anticoagulant activity higher than the original aptamer and TBABM a promising cytotoxic activity against breast and prostate cancer cell lines. These data suggest that the largely enhanced thermal stability and the retained G4 topology may concur to improve protein–G4 interactions, with the exclusion of possible minor local structural variations, which could contribute negatively as in the case of TBABL. Concluding, in this investigation, we proposed an unprecedented combination of different modifications of TBA G-tetrad core that have allowed an encouraging improvement of both the anticoagulant (in the case of TBAB) and antiproliferative activity to the disadvantage of the anticoagulant one (in the case of TBABM). Therefore, the opposite site-specific substitution of the G-core residues of TBA with commercially available nucleoside analogues appears as a positive approach to modulate the anticoagulant activity and the antiproliferative properties of this aptamer, increasing the possibility of developing new properly modified therapeutic oligonucleotides.

## Figures and Tables

**Table 1:** Name, sequences, T_m_, and ΔT_m_ of the studied ODNs. **B**, **L**, and **M** indicate 8-bromo-2′-deoxyguanosine, locked nucleic acid guanosine, and 2′-O-methylguanosine, respectively. See Materials and Methods for experimental details.

Name	Sequences (5′-3′)	T_m_ °C (±0.5)	∆T_m_ (°C)
**TBA**	GGTTGGTGTGGTTGG	52.0	-
**TBAB**	BGTTBGTGTBGTTBG	84.0	+32.0
**TBAL**	GLTTGLTGTGLTTGL	36.8	−15.2
**TBAM**	GMTTGMTGTGMTTGM	41.8	−10.2
**TBABL**	BLTTBLTGTBLTTBL	74.0	+22.0
**TBABM**	BMTTBMTGTBMTTBM	71.4	+19.4

## Data Availability

Data is contained within the article and Supplementary Material.

## Supplementary Materials

The following supporting information can be downloaded at: https://www.mdpi.com/article/10.3390/ijms26010134/s1.
