# Supplementary material for: Probing the Effects of Chemical Modifications on Anticoagulant and Antiproliferative Activity of Thrombin Binding Aptamer
Source: Int J Mol Sci. 2024 Dec 27;26(1):134. doi: 10.3390/ijms26010134 (PMC11719963; doi:10.3390/ijms26010134)
Supplement: Supplementary file 1 [file ijms-26-00134-s001.zip › ijms-3303846-supplementary.pdf]

Supplementary Material

Probing The Effects of Chemical Modifications on Anticoagulant and Antiproliferative Activity of Thrombin Binding Aptamer

Antonella Virgilio, Daniela Benigno, Carla Aliberti, Ivana Bello, Elisabetta Panza, Valentina Vellecco, Mariarosaria Bucci, Veronica Esposito* and Aldo Galeone.

Department of Pharmacy, University of Naples Federico II, Napoli, Italy.
* Corresponding author: VE: verespos@unina.it.

Table of contents

CD melting and annealing profiles
MTT assay at 24 h on cancer cell lines
Nuclease Stability assay
HPLC traces of TBA and its analogues
1H-NMR spectra of purified oligomers in D_2O at 80°C

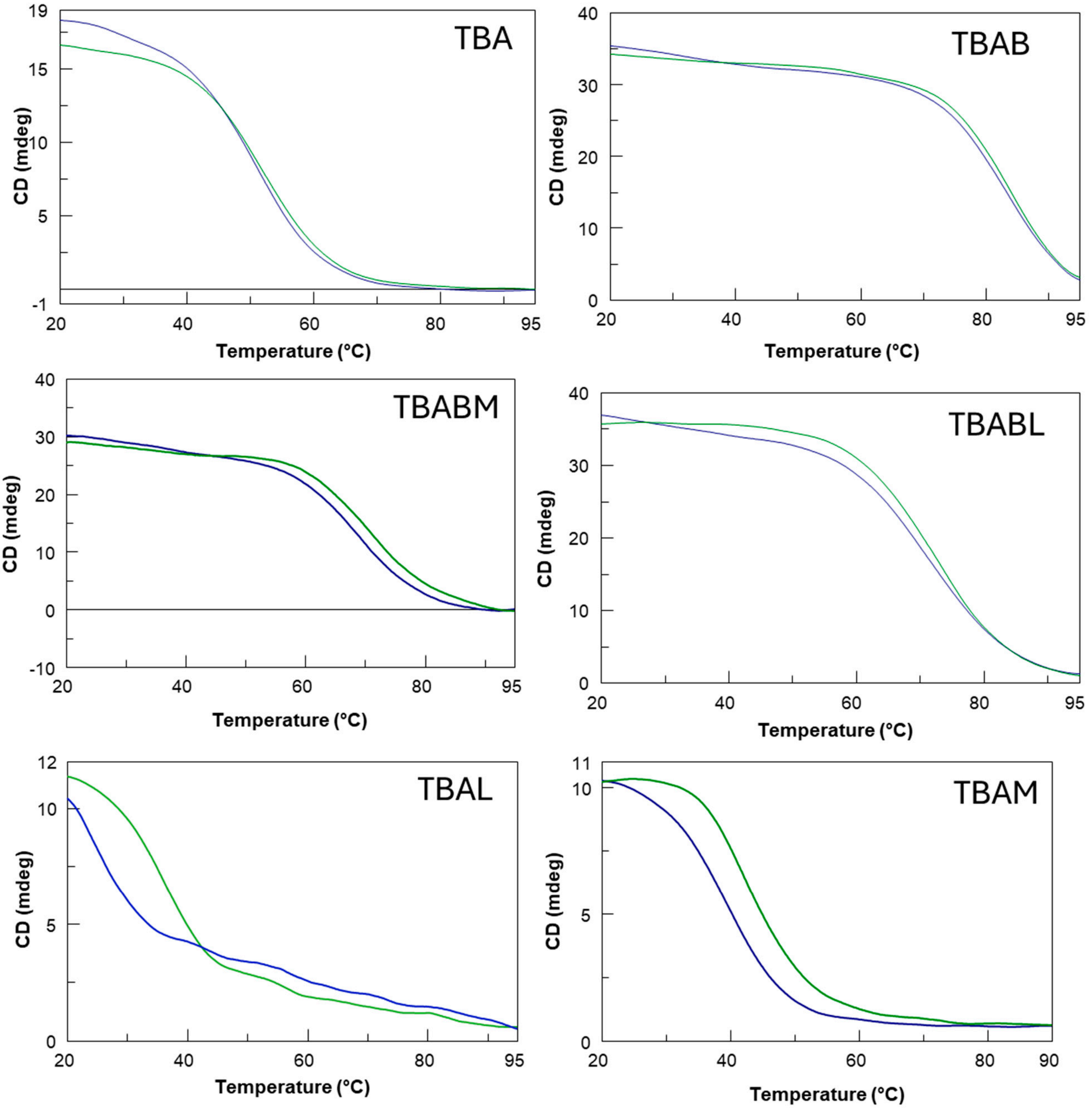

Figure S1. CD melting (green) and annealing (blue) profiles of the G-quadruplex formed by TBA and its investigated derivatives. See Materials and Methods for experimental details.

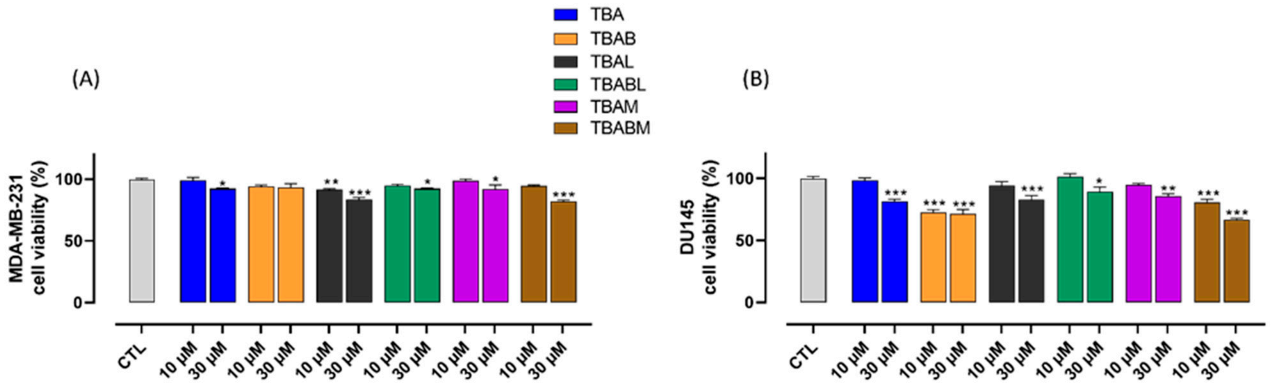

Figure S2. Effect of TBA and its analogues on MDA-MB-231 and DU145 cell proliferation. Cell proliferation was measured using the MTT assay and evaluated at 24 h. Each experiment (n = 3) was run in quadruplicate. * $p < 0.05$; ** $p < 0.01$; *** $p < 0.001$ vs. CTL.

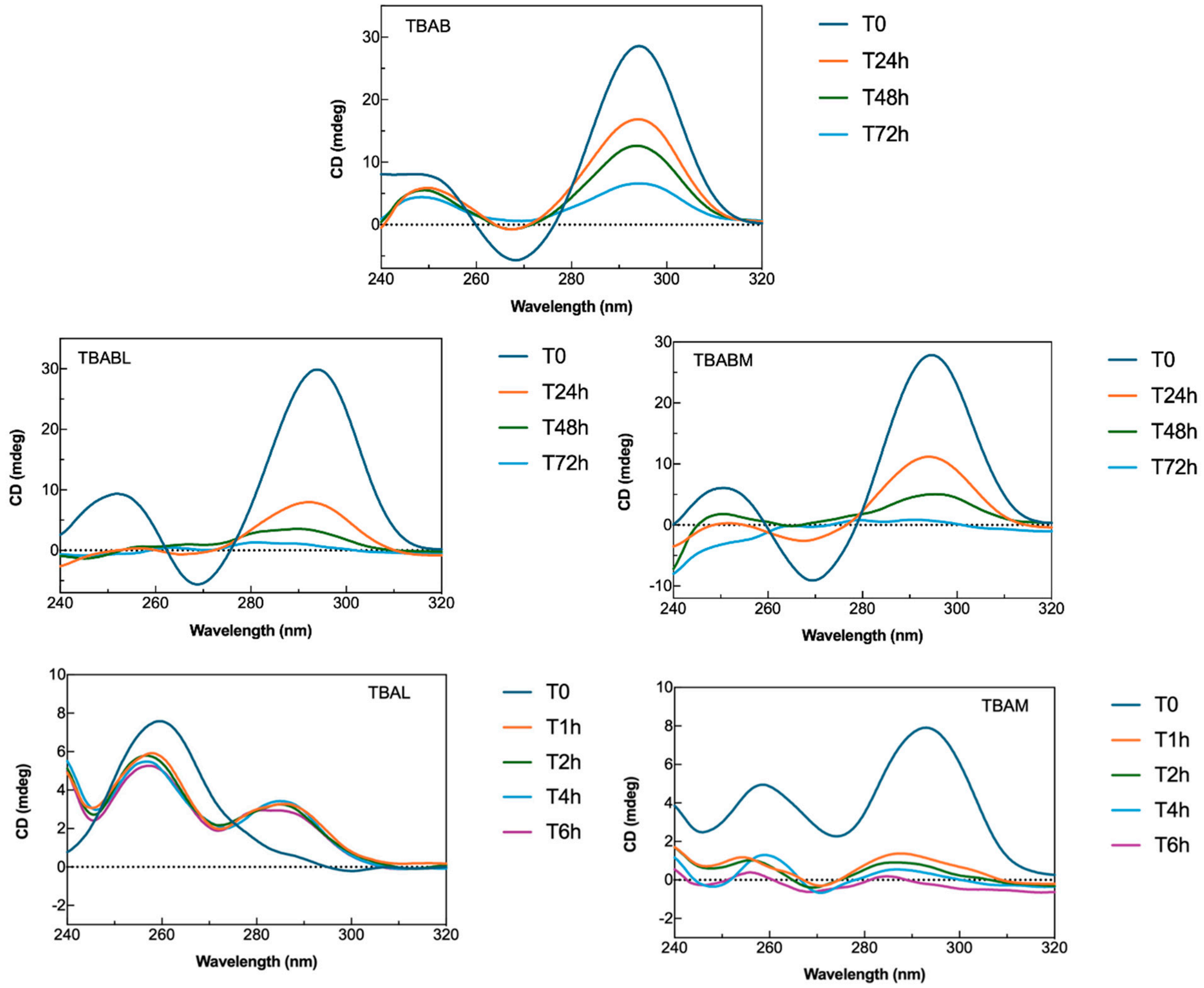

Figure S3. CD spectra of investigated ODNs in 10% Fetal Bovine Serum (FBS) diluted with Dulbecco's Modified Eagle's Medium (DMEM) and registered at different times at 37 °C. See the main text and the Section 4 for details.

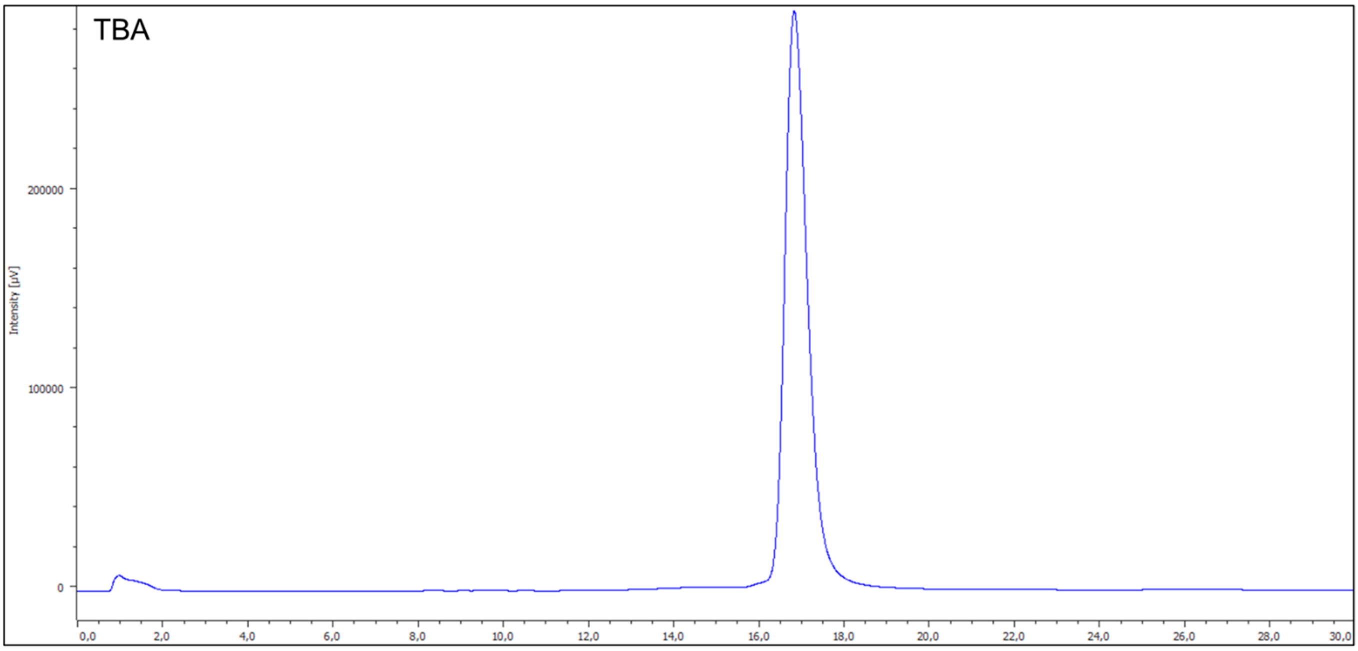

Figure S4. HPLC chromatograms of TBA and its analogues. See experimental section for details.

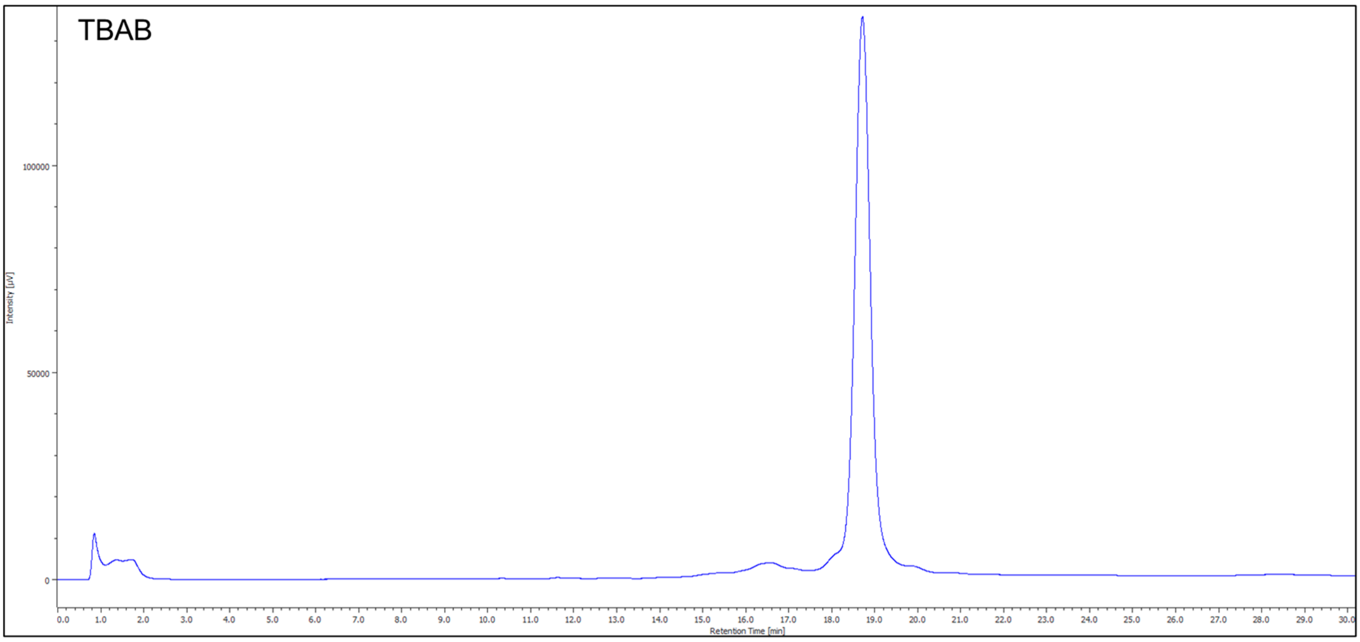

Figure S4. Cont.

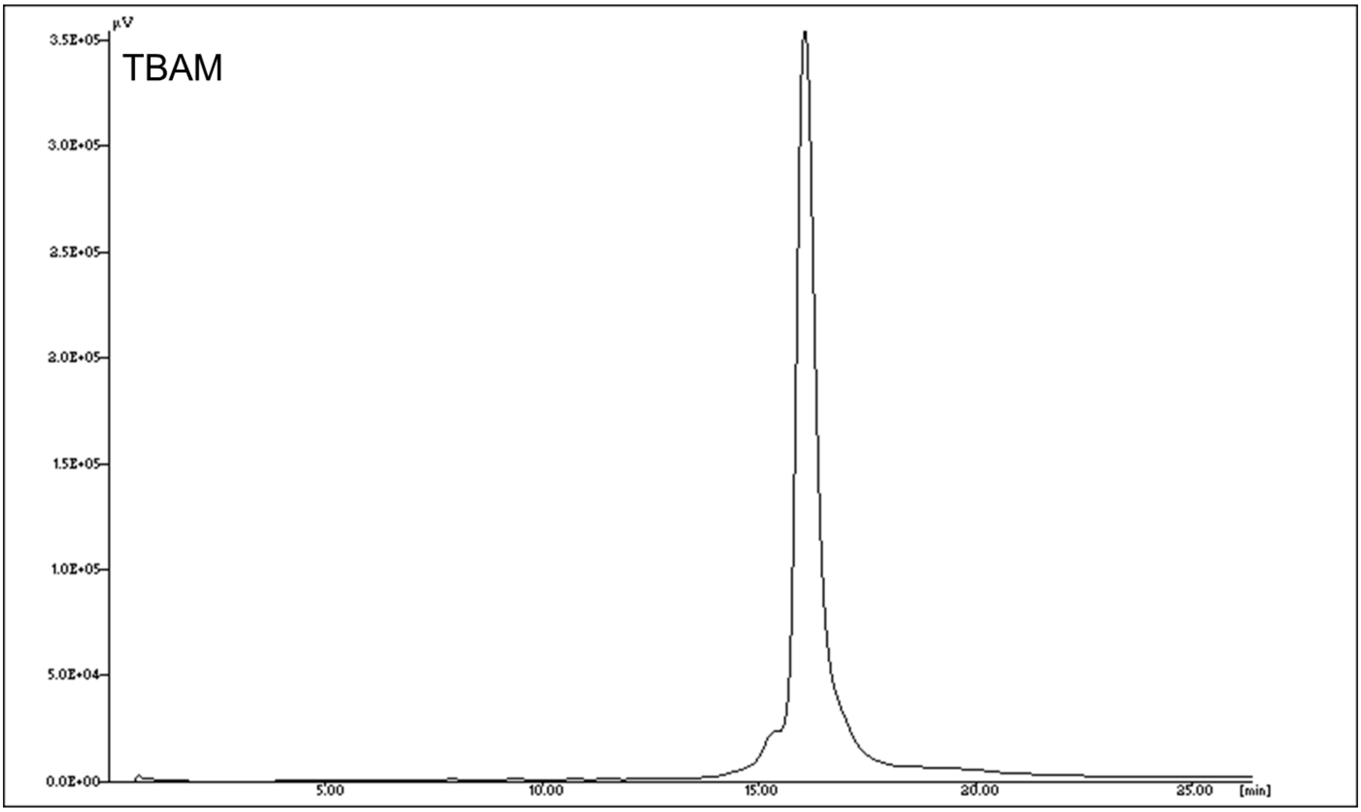

Figure S4. Cont.

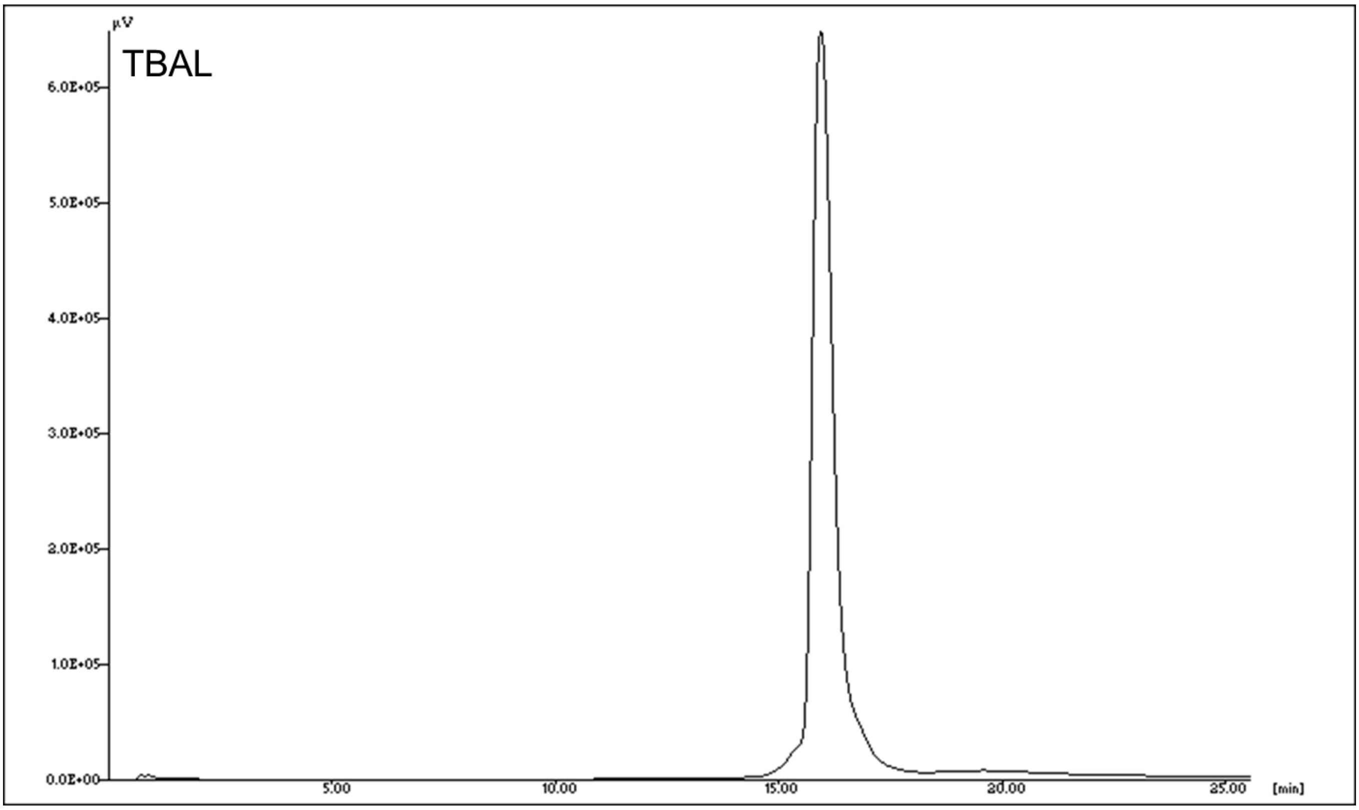

Figure S4. Cont.

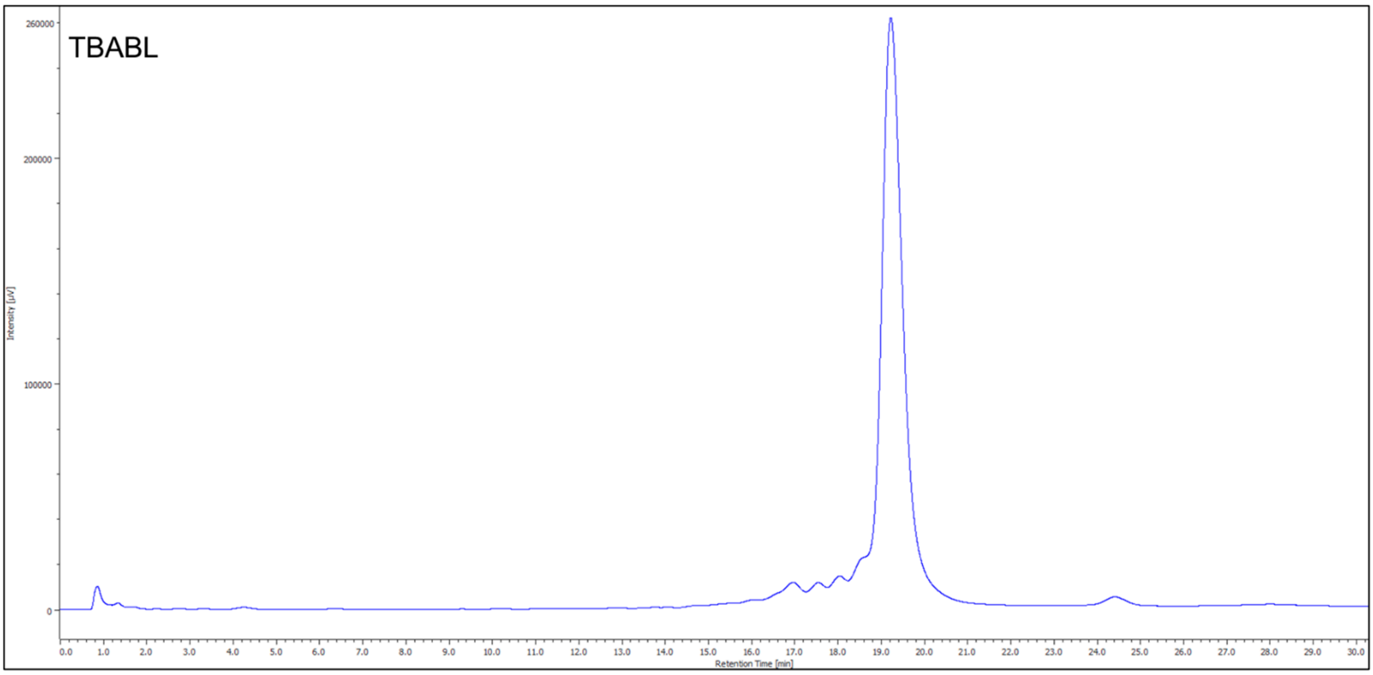

Figure S4. Cont.

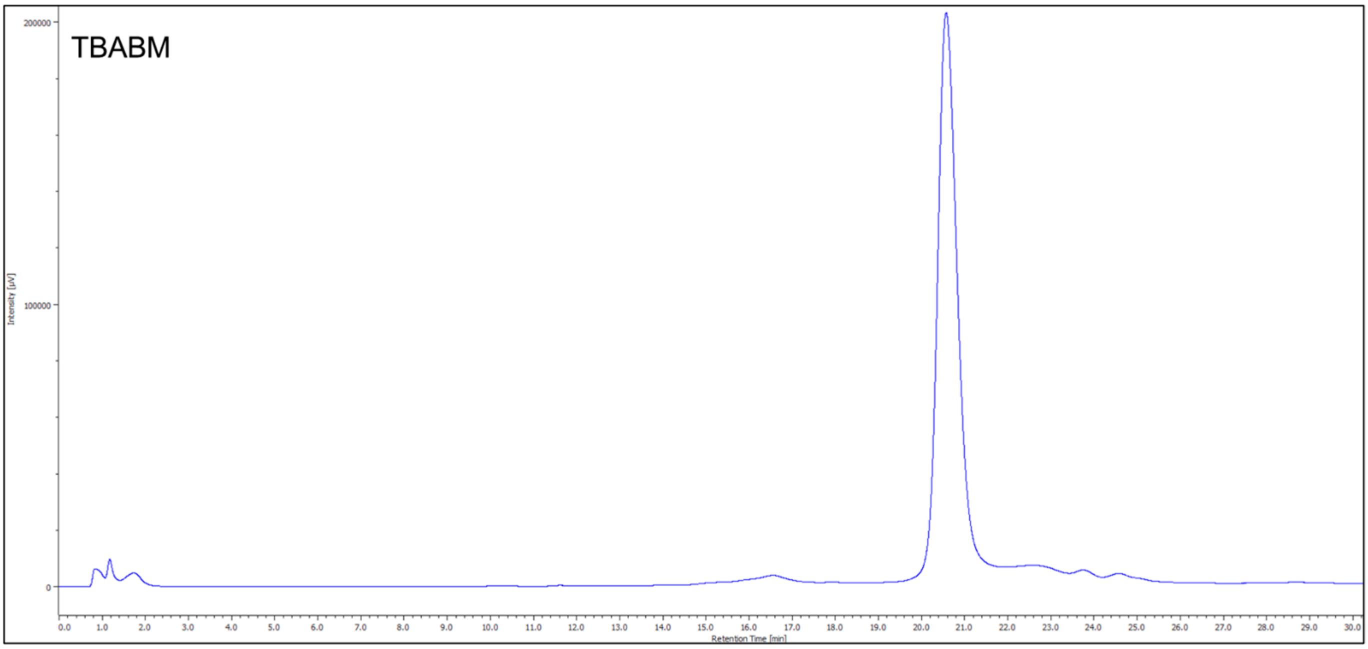

Figure S4. Cont.

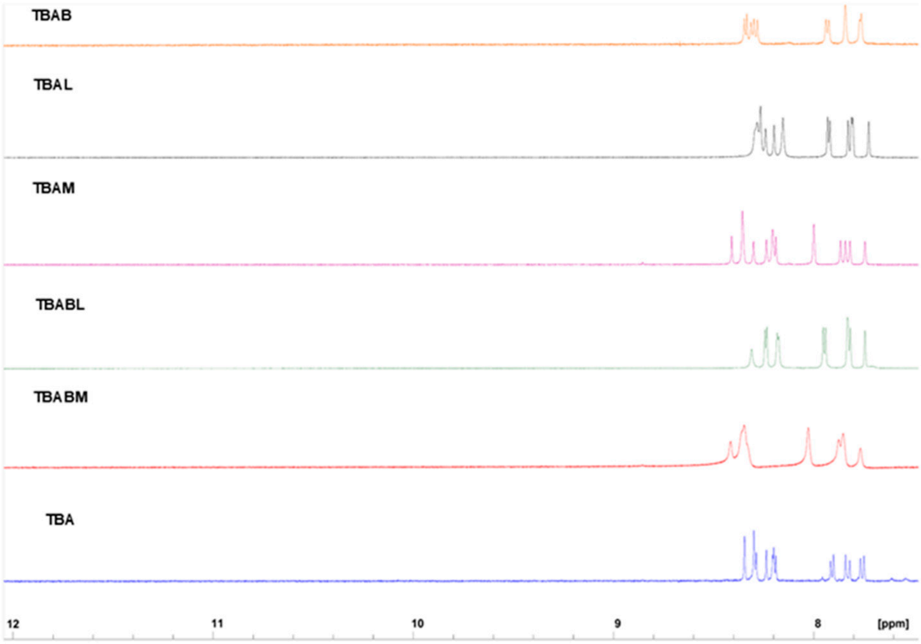

Figure S5. Aromatic region of the high-resolution NMR spectra (700 MHz, D_2O, 80°C, no salt) TBA and its investigated derivatives. See Materials and Methods for experimental details.
